# How E-Learning Environmental Stimuli Influence Determinates of Learning Engagement in the Context of COVID-19? SOR Model Perspective

**DOI:** 10.3389/fpsyg.2021.584976

**Published:** 2021-03-31

**Authors:** Junhui Yang, Michael Yao-Ping Peng, ShwuHuey Wong, WeiLoong Chong

**Affiliations:** ^1^Foreign Languages Institute, Fuzhou University of International Studies and Trade, Fuzhou, China; ^2^School of Economics and Management, Foshan University, Foshan, China; ^3^Department of Education, New Era University College, Kajang, Malaysia

**Keywords:** COVID-19, e-learning, environmental stimuli, learning engagement, S-O-R model

## Abstract

The COVID-19 pandemic at the beginning of 2020 has changed the conventional learning mode for most students at schools all over the world, and the e-learning at home has become a new trend. Taking Chinese college students as the research subject and drawing on the stimulus–organism–response (S-O-R) model, this paper examines the relationship between the peer referent, perceived closeness, and perceived control and the learning engagement. Using data from 377 college students who have used e-learning, this study shows that perceived closeness, perceived control, and peer referents in e-learning have a positive effect on the self-efficacy and well-being of students, thus improving students’ enthusiasm for learning. Our intent is to assist researchers, instructors, designers, and others in identifying effective methods to conceptualize and measure student engagement in e-learning.

## Introduction

Learning environmental stimuli have always been the key factor affecting the level learning intention among students (Chang et al., 2008). Many studies have shown that good learning environmental stimuli enhance students’ intrinsic learning motivation and help them to obtain the required knowledge and skills, thus achieving the scheduled goals (Bojuwoye et al., 2014). Mashau (2000) holds that schools have the responsibility for creating a favorable learning environment to promote effective learning, and students would benefit from mutual assistance among peers, improved courses, and high-quality teaching strategies in such an environment (Hewson and Hewson, 1983). Nowadays, college education is facing multiple challenges, and encouraging student engagement in learning has become the focus of increasing attention (Ngidi and Qwabe, 2006). However, most previous studies have explored the learning effect of e-learning taken by students in a stable environment. For instance, Al-Rahmi et al. (2019) adopted the technology acceptance model to explore behavioral intention of students in using the e-learning system. Abuhassna et al. (2020) established a research model with transactional distance theory and Bloom’s taxonomy theory and discussed the effect of relevant factors on learning satisfaction and academic achievements when they are involved in online learning platforms. Despite these studies showing that students who adopt SNSs for learning have significantly positive output, the situational context of the study design tends to be stable and provides students with e-learning environment in a guided manner (Jayachithra, 2020). For students who have to adopt e-learning due to the situational context extended by sudden environmental changes, the gap in their psychological cognition may lead to differences in learning effect and learning process (Shahzad et al., 2020). Thus, to understand this gap, this study further explores the psychological cognitive process in which students are involved in e-learning in the context of pandemic outbreak.

Allen and Seaman (2011) point out that about one third of students pursuing higher education in America have attended one or more e-learning subjects at home. Bach et al. (2006) regard e-learning as a critical component for the popularization of higher education. In particular, since the onset of the coronavirus disease 2019 (COVID-19) global pandemic in January 2020, countries all over the world have suspended (to a certain extent) economic, tourism, and catering activities, especially educational activities. In China, which suffered early from the epidemic, schools carried out remote teaching through e-learning to allow students to learn at home, and e-learning changes traditional educational activities to a digital form in terms of content and systems (Putra et al., 2019). However, students’ engagement in e-learning is obviously lower than in face-to-face teaching (Dietz-Uhler et al., 2007). Jonassen et al. (2003) propose that one possible reason may be that the student attention in e-learning is easily distracted by other factors. The change of the learning environment may also lead students to drop out of school or reduce their enthusiasm for pursuing their degree courses (Jaggars, 2014). Nevertheless, there are still many scholars who believe that e-learning brings positive effects to student learning (Al-Rahmi et al., 2019; Abuhassna et al., 2020; Jayachithra, 2020), particularly Shahzad et al. (2020) who explored the effect of COVID-19 in e-learning on students. Despite their studies showing that gender differences have different results for the use satisfaction, the relationship between the variables is significantly positive. The possible reasons come from the students’ learning autonomy and the establishment of learning targets. Besides, the changes in the learning environment brought by the COVID-19 global pandemic have changed students’ learning patterns, but scholars believe that learning targets still exist in their learning process. According to the social cognitive theory from Bandura (1986), as for the behavioral motivation derived from the establishment of learning targets, it does not come from the establishment of learning targets itself, but is influenced by the self-regulated process. Zimmerman (2008) also stated that self-regulated learning is a process of transformation, in which students will adjust their learning patterns and learning targets according to different situations and conditions. In other words, based on the self-regulated view, students are changed from traditional classroom learning to online learning, and at the moment, they will adjust the learning devotion mode and targets through the ways of orientation, regulation, persistence, and evaluation (David et al., 2007). Based on the above arguments, this study aims to explore the development process of learning engagement for students in the context of online learning.

Although students can acquire valuable knowledge and information from teachers *via* e-learning, it is not certain that such knowledge and information can be converted into high-quality competence. In studies of organizational management, Zaheer and Bell (2005) found that more attention should be paid to the internal capability of an organization in knowledge absorption (i.e., the learning engagement), while the acquisition of external knowledge and information tends to be more often discussed (Cohen and Levinthal, 1990). In terms of the connectedness stressed by Fabrizio (2009), students should be able to engage in learning actively, and transmit and use knowledge. Although teachers have provided abundant and valuable information in the context of e-learning, learning quality is particularly difficult to control (Putra et al., 2019) and a higher learning engagement from students is required. Most previous studies have discussed the importance of e-learning (Wang and Wang, 2011), but there have been few studies on the impact of the learning environmental stimuli perceived by students in e-learning on student engagement. When discussing the process of student learning engagement derived from the stimulation of learning environment, previous psychologists put forward the S-R theory (Hommel, 1997; Gast and Rothermund, 2011; Hazeltine and Schumacher, 2016). The S-R theory argues that an individual will directly make the right response after receiving external stimulus. In the past, most in the SO theory discussed how to transform into a specific response under given stimulating conditions, or the subsequent behavior of an individual after participating in a certain stimuli event (Hommel, 1997; Hazeltine and Schumacher, 2016). Since humans are organisms that generate psychological elements of feelings, moods, emotions, or attitudes in response to stimuli, thus the stimulus–organism–response (S-O-R) model is extended (Khan et al., 2017; Kim and Park, 2019; Zhai et al., 2020). To explore the psychological feelings and learning responses of students stimulated by the changes in learning environment under the circumstances of COVID-19, here, we introduce the S-O-R model and connect learning engagement in e-learning with the e-learning environment. Although previous studies have used this framework to examine the effect of the e-learning human–computer interface (HMI) on student behavior (Zhang et al., 2014, 2015), this study emphasizes the environmental factors of e-learning, rather than the technical factors, demonstrating the applicability of using the S-O-R model when considering learning engagement in the context of e-learning. In particular, this study focuses on the current situation of learning engagement among college students in the context of e-learning due to the COVID-19 pandemic.

## Literature Review and Hypotheses Development

### Theoretical Background

The S-O-R model involves three components: stimulus, impact, and response. It assumes that stimuli (S) are included in the external environment and cause changes to people’s internal organisms (O), which in turn affect their behavioral responses (R; Mehrabian and Russell, 1974). This model is used to conceptualize the individuals’ responses to environmental information. It can capture behavioral responses and elements in complex decision-making processes (Bagozzi, 1983). In this theoretical framework, stimuli appear in different forms, including environmental factors and interpersonal relationship (Animesh et al., 2011; Liu et al., 2016). Such changes to internal status appear in the perception of stimuli and behaviors, including affective, cognitive, perceptual, and mental activities. Zhai et al. (2020) argued in the study that when discussing students’ engagement in e-learning or SNSs, the S-O-R model is a good fit for the context used in researching the online user behaviors. With the spread of the global COVID-19 pandemic, many patterns of learning have begun to transform from offline classroom to online classroom, and the sudden changes in learning environment have compelled students to try to adopt multimedia tools for learning (Khan et al., 2017; Zhan et al., 2020). The psychological changes may induce students to have different learning styles and engagement behaviors; thus, it is necessary to utilize the S-O-R model to further explore the development of their entire learning process.

Previous studies of the S-O-R model have considered a wide range of stimuli, including social support (Zhang et al., 2014), flow (Animesh et al., 2011; Gao and Bai, 2014; Zhang et al., 2014), feeling (Koo and Ju, 2010; Vieira, 2013; Kim and Johnson, 2016), and interaction (Mollen and Wilson, 2010; Animesh et al., 2011; Zhao and Lu, 2012). The individual response after the reception of stimuli refers to the effective attitude and intention in subsequent individual behaviors, such as learning autonomy and learning intention (Animesh et al., 2011; Ha and Im, 2012). When it comes to the application of models, scholars set up different S-O-R models according to the situational conditions, making the application of the S-O-R model more perfect. For instance, Zhan et al. (2020) adopted the S-O-R model to explore how privacy concern develops knowledge hiding perceptions of the learners, thereby affecting their online collaboration. Good reliability, validity, and model fits are shown in their study, such as χ^2^ (CMIN/*df*) = 2.56, *p* < 0.001, standardized root mean square residual (SRMR) = 0.06, IFI = 0.91, CFI = 0.92, TLI = 0.91, and RMSEA = 0.074. Khan et al. (2017) established the S-O-R model to study the students’ flow experience in education and other personality traits, and showed that the research model also has good model fits, such as χ^2^/*df* = 2.16, CFI = 0.969, TLI = 0.940, and RMSEA = 0.060. Thus, it can be proved that constructing student learning status with the S-O-R model can gain a good explanation.

According to the S-O-R model, all behavioral outcomes involve a process of integrating of internal and external parts: the external interactive process of people with the environment and the internal psychological process of acquisition and obtainment (Illeris, 2003). The external interactive process is the social dimension, such as perception and contact. It affects the integration of environment and individuals and changes people’s behaviors. We believe that the stimuli perceived in the e-learning environment can be considered stimuli of the external environment and are correlated with the mental response generated in learning (subjective well-being) and self-efficacy. We therefore consider here how peer referent, perceived closeness, and perceived control are related to students’ mental response. An internal psychological process is a process where the cognitive function and subjective feeling interact with each other (Illeris, 2003), and here, we divide the internal psychological process into two parts: self-efficacy and subjective well-being.

### Learning Engagement

Learning engagement is the student behavior of participating in learning activities for better acquiring knowledge or skills (Hu and Hui, 2012), and it is susceptible to the qualities of the learning environment. Learning engagement emphasizes the importance of behavior (e.g., engagement), affection (e.g., well-being or satisfaction), and cognitive engagement in learning (Lam et al., 2012). It is one of the foremost factors for improving learning outcomes, as shown by many previous studies (Klem and Connell, 2004; McMahon and Portelli, 2004; Carini et al., 2006).

When students engage in learning on their own initiative, they take initiative in and/or concentrate on acquiring and applying new skills or knowledge, solve problems using underlying approaches, and show a positive attitude toward their learning process (Deater-Deckard et al., 2013). The development of models and measures that promote student learning engagement is crucial to the development of the field of education (Zahn, 1980). The more students engage in learning, the higher their enthusiasm for learning will be and the better progress they will make.

### Self-Efficacy

Scholars argue that an individual’s behavioral outcome is affected by environmental factors, in particular situation (Dinther et al., 2011), especially for those beliefs leading to success. This belief is called “self-efficacy” and it is an important cognitive variable used to explain personal factors in individual formative behavior and interactions with the environment (Lent et al., 2014; Sheu et al., 2014). Self-efficacy has been widely applied in the field of education to discuss students’ psychological cognitive factors and the positive influence of their learning performance on career development. Contemporary studies hold that more research on the relationship between self-efficacy and learning performance improvement needs to be carried out (Tims et al., 2014). Tims et al. (2014) assert that when individuals have a high level of self-efficacy, they make more effort to obtain learning-related resources that can help them engage more deeply in learning (Bakker and Demerouti, 2014). It can thus be deduced that when students have a high level of self-efficacy, their learning engagement may be further improved. Based on the above, this study makes the following hypothesis:

H1: Self-efficacy is positively correlated with learning engagement.

### Subjective Well-Being

Subjective well-being is consistent well-being or satisfaction that allows individuals to feel successful and deal with life pressure (Diener and Seligman, 2004; Huppert and So, 2013). Students’ subjective well-being often involves the quality of school teaching and a positive emotional and cognitive evaluation of the school (Scrimin et al., 2016). Subjective well-being is critical to successful learning engagement among college students, because it promotes active learning, critical thinking, optimal performance, learning participation, and physical and mental health (Huppert and So, 2013; Steptoe et al., 2015). Given the situation resulting from the COVID-19 pandemic, in which colleges and universities in mainland China have been using e-learning instead of traditional in-person teaching models for an extended period of time, students can perceive the more informal environment and are more self-centered (Tremayne et al., 2008). Student well-being has a positive impact on accepting new knowledge, facing new challenges, and maintaining learning motivation. Therefore, this study makes the following hypothesis:

H2: Subjective well-being is positively correlated with learning engagement.

### Perceived Control

Teaching classroom control is often defined as a single dimension ranging from teacher control to student autonomy, as well as teacher and student control of learning (DeCharms, 1976). Classroom control depends on the teaching content and direction, as controlled by teachers, and the opportunities for self-directed learning for students (Bandura and Wood, 1989). Connell (1985) holds that perceived control could be improved by providing students with opportunities for choice and self-directed learning. Pintrich and de Groot (1990) believe that learning outcomes depend on students’ view of organized teaching, their specific learning goals and clear explanations. Learning autonomy is the major determinant in ensuring sustainable self-control and improving learning performance (Connell, 1985; Pintrich and de Groot, 1990; Ames, 1992). There is no conflict, however, between giving more decision-making power to students in class and preserving the teaching responsibilities of teachers (Randi and Corno, 2000). Eckles (1993) found that low sense of control among students may have an adverse effect on their intrinsic motivation and academic performance. The expectation for gaining higher levels of inner achievement is therefore related to a higher degree of student control. When teachers do not share classroom decisions with students—that is, in teaching based on teacher control—students tend to avoid self-regulating strategies (Ryan and Grolnick, 1986; Eccles et al., 1993). Students who think they have no substantial control over their learning will show a lower level of response. Giving students the opportunity to make choices can enhance their intrinsic motivation and their engagement in learning (Ryan et al., 1985; Zimmerman and Kitsantas, 1999). We can thus infer that students have the highest self-recognition and self-efficacy when they perceive the classroom environment as being mainly student controlled. We thus propose the following hypothesis:

H3: Perceived control is positively correlated with self-efficacy.

According to theoretical and empirical research, perception of autonomous control promotes a higher level of well-being, because respondents with autonomous control can easily meet their basic psychological needs (Deci et al., 2001; Deci et al., 2006; Deci and Ryan, 2008). Decades of research have shown that perceived control is associated with motivation and various happiness indexes, such as the parent–child relationship (Clark and Ladd, 2000; Abad and Sheldon, 2008), and friendship (Demir et al., 2013). This idea has also been applied to the teacher–student relationship: students’ perceived control has been shown to promote the internal perception of learning (Sheldon and Krieger, 2007; Filak and Sheldon, 2008; Overall et al., 2011; Bonneville-Roussy et al., 2013; Simon et al., 2015). For example, in a study on law school students, Sheldon and Krieger (2007) show that students who believe teachers support their choices in the classroom have a higher GPA, higher ABA scores, and more motivation for hunting jobs after graduation. In another study, Black and Deci (2000) show that the adoption of teaching strategies to promote student autonomy in class can support student success. Specifically, the students’ perception of autonomous control from the teacher indicates inner satisfaction, increase of learning time, and a more comprehensive command of the entire curriculum knowledge. Based on this, we suggest the following hypothesis:

H4: Perceived control has a positive effect on subjective well-being.

### Perceived Closeness

Perceived closeness is the sense of mutual trust and understanding generated from frequent interpersonal communication and pleasant interaction (Carey, 1986; Gremler and Gwinner, 2000; Frisby and Martin, 2010; Ryan et al., 2011). When being applied in the relationship between teachers and students, it is interpreted as “the results of communication with teachers perceived by students” (Frisby et al., 2014). Student self-efficacy in the teaching process depends on the individual characteristics of the teacher (Rots et al., 2002). Studies have shown that students have the greatest motivation when they perceive a positive correlation with teachers (Connell and Wellborn, 1991; Skinner and Belmont, 1993; Ryan and Deci, 2000). The relationship between students and teachers is an important predictor of subjective well-being (Aelterman et al., 2007). Building a strong supportive relationship with teachers makes students feel safer, more secure, and more capable in the school environment, thus affecting intrinsic motivation (Olivier and Archambault, 2017). The behavioral motivation of students is closely related to the teacher’s ability to encourage cooperation. Such cooperation will also affect the teaching results, including the students’ perception of themselves in class. This perception is obvious, especially in collective communication (McCroskey et al., 2006). Therefore, this study makes the following hypothesis:

H5: Perceived closeness has a positive effect on self-efficacy.

School is, in essence, a place of relationships. In this case, interpersonal relationships can have a positive or negative effect on student well-being (Bernard et al., 2007; Redmond et al., 2013). Kristja’nsson (2007) believes that affable people help to increase well-being by demonstrating lovely, positive, and good manners. Students value teachers who try hard to build relationships with them (Pomeroy, 1999; Sellman, 2009) and those who are “affable, smart, and interesting” (Hutchings et al., 2008). Teachers can thus carry out effective practice in class by promoting teacher–student interaction and striving to support students’ social and affective functions. Studies have shown that students’ well-being score is very low if their teachers show uncertainty and dissatisfaction when students make presentations in class (Stevens and Sanchez, 1999; Holfve-Sabel, 2008). As Barton et al. (2000) emphasize, “Students generally spend a lot of time in school, and the quality of experience during the period of time with teachers is bound to affect emotional health.” In the process of school teaching, students who are more emotionally connected with teachers show positive development trajectories in society and the academic sector (Ryan et al., 1994; Harter, 1996; Ladd et al., 1999; Roeser et al., 2000; Hamre and Pianta, 2001). This can also be extended to a more extensive educational background. For example, in an e-learning environment, teacher–student closeness will affect student well-being. Therefore, this study makes the following hypothesis:

H6: Perceived closeness has a positive effect on subjective well-being.

### Peer Referents

Studies on point-to-point social impact in the student learning environment have shown that student characteristics and behavior tend to concentrate spatially and temporally (Aral and Walker, 2011). The mechanism for this is generally considered to be peer influence or peer referents (Turner, 1991). Some scholars have proposed that people will be positively or negatively evaluated based on the compliance of their behaviors to their role and surroundings (Eagly and Karau, 2002), so the influencing process on interactive behavior between peers cannot be ignored. Studies in the field of interpersonal relationships have repeatedly shown that peer referents play an important role in influencing perception and behaviors; the more individuals observe peers engage in a certain behavior, the more likely they are to engage in the same or similar activities (Beams et al., 2003; Bapna and Umyarov, 2015). If students observe that many peers are engaging in e-learning, they are more likely to engage in the same (Chen, 2008). In terms of online herd behavior, more peer referents tend to increase students’ perception of themselves, and students further believe that their behaviors are recognized by their peers. Based on the above arguments, we make the following hypothesis:

H7: Peer referents have a positive effect on self-efficacy.

In a learning environment, peers have an obvious internal influence and may have an important influence on subjective well-being. Albanesi et al. (2007) propose that relationships between peers can predict students’ social well-being, because references between peers are intuitive for students. For example, if most of your friends agree about an idea, you may feel obliged to show your agreement. When an individual compares his or her own behavior with that of a friend, subjective well-being will occur if his or her behavior is recognized by that friend (Lord and DeZoort, 2001). In e-learning lectures, if the understanding of knowledge a college student possesses is recognized by peers, a positive peer referent is generated. These referents are associated with student perception of well-being in the learning process. Grobecker (2016) believes that peer support has a positive impact on students’ learning, motivation, and confidence, so it has a bearing on students’ subjective well-being. All of these arguments indicate that peer referents may be an important prerequisite for perceiving well-being. Based on the above arguments, we make the following hypothesis:

H8: Peer referents have a positive effect on subjective well-being.

**FIGURE 1:**
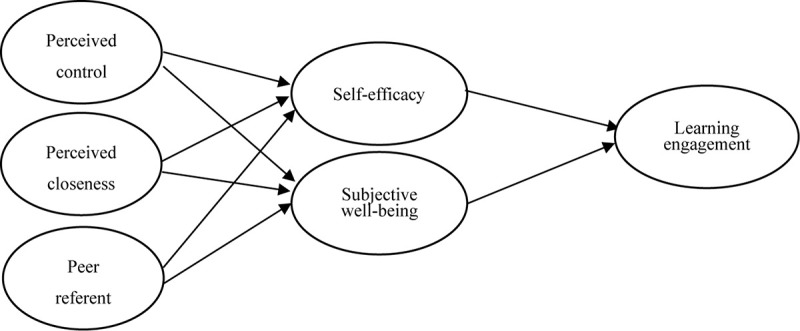
Research framework.

The S-O-R model can explain the willingness of students to engage in learning on their own initiative in the context of e-learning. To include the e-learning environment in the model as social environment stimuli, we assume that perceived control, perceived closeness, and peer referents are positively correlated with the self-efficacy and subjective well-being of students, which in turn affects learning engagement in the e-learning context. Based on this, we have interconnected the set of theoretical constructs in the S-O-R model and elucidated the underlying mechanisms in the research model (Figure 1).

## Methodology

### Data Collection

To contain the further spread of COVID-19, all colleges and universities in Mainland China have replaced traditional face-to-face teaching with e-learning in 2020. Since the purpose of this study is to explore the learning process and engagement of students brought by the changes in learning environment under the circumstances of the COVID-19 pandemic, purposive sampling is adopted to collect samples in line with this study. To make the samples meet the demands of the research model and research purpose, several conditions were set up in the sampling process. Firstly, the students should have the experience of offline classroom learning in the college and were not about to graduate, so the senior students were excluded and the sophomore and junior students were retained. Secondly, the multimedia tools used by students in online learning were mostly laptops or iPads, excluding students who use mobile phones. Thirdly, the hours for students taking multimedia tools for online learning should be at least 20 h per week. With the above three conditions, college students were taken as samples. This study takes college students as the research subject, selected four universities in Mainland China, and distributed 500 questionnaires. Questionnaires were distributed and collected in May and June 2020, and a total of 422 questionnaires were received. Of these, 45 questionnaires were eliminated due to questions that were not answered, so the valid questionnaires numbered 377, resulting in a valid response rate of 75.4%. Male students accounted for 46.8% of the respondents and female students for 53.2%; freshmen accounted for 35.6%, sophomores for 31.2%, and juniors for 33.2%; students majoring in social science accounted for 56.1%, and students majoring in natural science for 44.9%; students from public universities accounted for 69.4%, and students from private universities for 31.6%.

### Instrument

To test the research model, a survey instrument was developed with each construct measured using multiple items. Most items were adapted from existing measures in the related literature with confirmed content validity and reliability, and then modified to fit our research context. Perceived closeness was measured by three items adapted from Ng (2013). Peer referents were measured by three items adapted from Eckles et al. (2005). For perceived control, an extended form of the Student Decision-Making Scale (Eshel, 1991) was employed. This scale presented a student perspective on shared control in class. For self-efficacy, four items were selected on the basis of prior scale and item analyses of Asian applications. Subjective well-being was measured using Keyes’s (2005) subjective well-being instrument (adolescent version). All items were measured with a five-point Likert scale (1 = *totally disagree*; 5 = *totally agree*). All items of scales are shown in Table 1.

**TABLE 1:** Items of scales.

Variable	Items	Factor loadings
Perceived control	In the course of online learning, I can think independently.	0.868
	In the course of online learning, I can take control of the learning content.	0.879
	In the course of online learning, I can control my own pace.	0.817
	In the course of online learning, I have class autonomy.	0.841
Perceived closeness	In the course of online learning, I feel a sense of closeness with teacher.	0.777
	In the course of online learning, I feel a sense of intimacy with teacher.	0.879
	In the course of online learning, my interaction with the teacher is different from that in offline classroom.	0.789
	In the course of online learning, I think I can talk to my teachers about anything.	0.834
Peer referent	In the course of online learning, I feel valued when I do things my classmates do.	0.821
	In the course of online learning, I feel approved when I do things my classmates do.	0.744
	In the course of online learning, I feel more personally accepted when I do things my classmates do.	0.780
	In the course of online learning, I do operations similar to my classmates.	0.831
Self-efficacy	In the course of online learning, I am competent to solve the problems of e-learning.	0.791
	In the course of online learning, when I come across problems, I can find solutions to them.	0.829
	I will try my best to achieve the online learning targets set by myself.	0.822
	I am well prepared to face and handle the demands of e-learning.	0.655
Subjective well-being	I feel cheerful.	0.872
	I find it interesting to study.	0.883
	I have confidence in my ideas and opinions.	0.876
	I think the society will be better.	0.763
Learning engagement	After taking course of online learning, I am willing to take the initiative to analyze problems.	0.848
	After taking course of online learning, I am willing to take effective learning.	0.863
	After taking course of online learning, I am willing to solve practical learning problems.	0.841
	After taking course of online learning, I am willing to engage in knowledge acquisition.	0.683

Because data were collected in China, translation and back-translation were adopted to ensure the translation quality. First, we consulted three professors of linguistics to understand the significance and readability of each item. The English questionnaire was then translated into Chinese with their help. Second, the Chinese questionnaire was translated into English by two Ph.D. candidates otherwise unconnected with this study. Third, we compared the translated items with the original items in English. To ensure the consistency of the two English versions, we improved the translation and eliminated all inconsistencies.

## Results

### Evaluation of the Measurement Model

In our data analysis, we used partial least squares (Smart PLS 3.0), a variance-based latent variable structural equation modeling (SEM) technique. The primary advantages of PLS-SEM include the relaxation of normal distributional assumptions required by the maximum likelihood method used to estimate models using CB-SEM, as well as PLS-SEM’s ability to easily estimate much more complex models with smaller sample sizes (Hair et al., 2019; Khan et al., 2019). The above reasons support the use of PLS at an appropriate SEM method for this study. Prior to evaluating the research model, we conducted several analyses to ensure that the latent constructs exhibited factorial validity and reliability. As shown in Table 1, all items show a high load for their relevant factors, but a low crossover load for other factors, indicating good convergence and discriminatory validity.

However, confirmatory factor analysis was carried out to evaluate reliability and validity. As shown in Table 2, Cronbach’s α values ranged between 0.779 and 0.873, and the complex reliability (CR) values ranged from 0.858 to 0.913. All values are higher than the threshold value of 0.7, showing an adequate reliability. Moreover, the average variance (AVE) ranged from 0.604 to 0.725, which is higher than the suggested threshold value of 0.5. This indicates sufficient convergent validity (Fornell and Larcker, 1981). Third, this study compares the square root of the AVE and structural dependence to test the discriminant validity (Gefen and Straub, 2005). As shown in Table 3, all dependencies are lower than the square root of the AVE, showing a sufficient discriminant validity.

**TABLE 2:** Measurement properties.

	1	2	3	4	5	6
1. Subjective well-being	0.850					
2. Peer referent	0.362	0.795				
3. Learning engagement	0.644	0.346	0.812			
4. Perceived control	0.483	0.303	0.420	0.851		
5. Perceived closeness	0.327	0.339	0.309	0.277	0.821	
6. Self-efficacy	0.532	0.473	0.466	0.424	0.265	0.777
α	0.807	0.806	0.826	0.873	0.838	0.779
AVE	0.722	0.632	0.659	0.725	0.674	0.604
CR	0.912	0.873	0.885	0.913	0.892	0.858

**TABLE 3:** Results of the hypotheses testing.

Hypotheses	Std. β	*t* value	Significance CI (2.50–97.5%)	VIF	*f*^2^
**Direct paths**					
H1: Self-efficacy → learning engagement	0.172**	3.316	(0.070∼0.276)	1.395	0.038
H2: Subjective well-being → learning engagement	0.553***	12.481	(0.461∼0.634)	1.395	0.388
H3: Perceived control → self-efficacy	0.298***	5.302	(0.188∼0.410)	1.144	0.113
H4: Perceived control → subjective well-being	0.381**	7.680	(0.056∼0.257)	1.144	0.183
H5: Perceived closeness → self-efficacy	0.059	1.140	(-0.038∼0.159)	1.174	0.004
H6: Perceived closeness → subjective well-being	0.156***	3.046	(0.056∼0.257)	1.174	0.030
H7: Peer referents → self-efficacy	0.363**	6.822	(0.257∼0.467)	1.194	0.161
H8: Peer referents → subjective well-being	0.194**	3.413	(0.084∼0.304)	1.194	0.045

**p < 0.01, ***p < 0.001.

### Testing Structural Model Fit

Before proceeding to examine the structural model, we first tested the model fit. Henseler et al. (2015) proposed three model fitting parameters: the SRMR, the normed fit index (NFI), and the exact model fit. According to Henseler et al. (2015), the evaluation standards for convergent validity are (1) NFI should be greater than 0.9, (2) SRMR should be less than 0.08, and (3) the exact model fit, which tests the statistical (bootstrap-based) inference of the discrepancy between the empirical covariance matrix and the covariance matrix implied by the composite factor model. Dijkstra and Henseler (2015) suggested the d_LS (squared Euclidean distance) and d_G (geodesic distance) as two different ways to compute this discrepancy. Henseler et al. (2015) indicated that dULS and dG were < the 95% bootstrapped quantile (HI 95% of dULS and HI 95% of dG). In this study, the SRMR value was 0.062 (<0.08), the NFI was 0.934 (>0.90), the dULS was < the bootstrapped HI 95% of dULS, and dG was < the bootstrapped HI 95% of dG, indicating the data fits the model well.

### Structural Model Analysis

**FIGURE 2:**
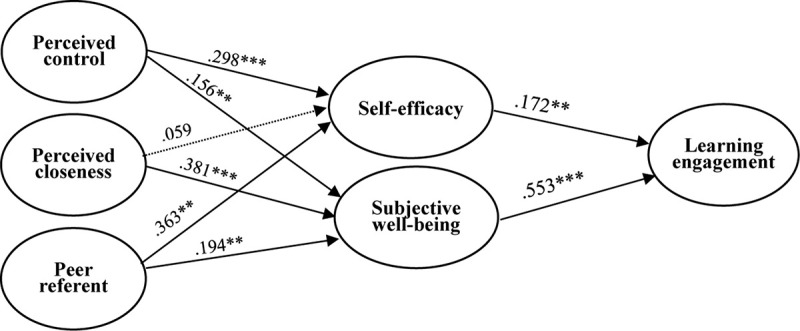
Structural model. ^∗∗^*p* < 0.01 and ^∗∗∗^*p* < 0.001.

We used the SRMS criterion to evaluate the model’s goodness of fit. In our examples, the SRMS is 0.062, lower than the 0.08 proposed by Henseler et al. (2016), indicating a satisfactory model fit. After evaluating that the measurement was satisfactory, we assessed the structural model. The hypotheses were examined by the percentage of variance explained and the significance of the structural paths. Figure 2 and Table 3 show the test result of the PLS analysis including control variables. Perceived control (β = 0.298, *p* < 0.001) and peer referents (β = 0.363, *p* < 0.001) are positively correlated with self-efficacy, so H3 and H7 are supported. However, perceived closeness (β = 0.059, *p* = 0.258) is not significant for self-efficacy, so H5 is not supported. Perceived control (β = 0.156, *p* < 0.01), perceived closeness (β = 0.381, *p* < 0.001), and peer referents (β = 0.194, *p* < 0.01) have a significantly positive correlation with subjective well-being; thus, H4, H6, and H8 are supported. Self-efficacy (β = 0.172, *p* < 0.01) and subjective well-being (β = 0.553, *p* < 0.001) are positively correlated with learning engagement, so H1 and H2 are supported.

## Discussion

The results of this study indicate that a harmonious relationship between teachers and students, peer referents among students, and student autonomous control over teaching contribute a lot to the learning engagement of students. With Chinese college students as the research sample, an empirical study was carried out to explore the dependence relationship between perceived closeness, perceived control, peer referents, self-efficacy and subjective well-being, and learning engagement using the S-O-R model. This study fills the theoretical gap in terms of research on student learning engagement in the e-learning context and will enhance theoretical generalizations.

Based on our findings, this study aims to make the following contributions. First, few studies have confirmed the influence of stimulating factors in e-learning environment on students’ learning engagement. This study used the COVID-19 pandemic as the research background, discussed learning engagement among college students in the long-term e-learning process, and attempted to provide practical inspiration for schools to carry out more e-learning practices in the future. Second, most of the previous studies on the S-O-R model focused on the importance of external environmental stimuli, but few studies have analyzed the role of specific factors in e-learning. This study aimed to fill this gap and enrich the applications of the S-O-R model. Third, in addition to verifying the research framework established by the S-O-R model, this study also focused on the perspective of e-learning. Our research findings will provide more insights and suggestions for e-learning management.

The results of this study show that perceived closeness, perceived control, and peer referents are positively correlated with subjective well-being. This signifies that, in the e-learning context, students’ participation in and control of the teaching process, their high closeness with teachers, and their mutual recognition and behavioral referents with peers will make students feel satisfied and thus produce learning-related well-being. The close relationship between teachers and students is one of the main factors affecting the psychological status of students, which is consistent with the results of previous research (Richmond et al., 2015; Rogers, 2015). Perceived control and peer referents are positively correlated with self-efficacy. In other words, students perceive higher self-efficacy when students think that the teacher has given them more freedom to make choices in class. In addition, the mutual effect and synchronized behavior of peers is also an external influencing factor that has a positive effect on students’ internal self-efficacy. This study also found that self-efficacy and subjective well-being are positively correlated with learning engagement. This is in line with prior findings; for example, Bandura and Wood (1989) stress the importance of self-efficacy for changing and using capabilities, which is one of the factors for improving academic performance. The results of this study are consistent with the view of self-learning, indicating that learners with learning characteristics of self-regulation have more positive and active learning styles, who can set practical and feasible learning targets according to their own learning, recognize available resources, choose proper learning strategies, and can evaluate their own learning achievements (Bembenuty, 2011).

However, we also found that the relationship between perceived closeness and self-efficacy was not supported. Previous studies have put forward that this close relationship is one of the most significant factors that influence courses (Richmond et al., 2015; Rogers, 2015). The influence of this kind is not necessarily positive. In e-learning, changes in the learning environment and a long stay at home inevitably cause learning difficulties. Thus, when teachers have a harmonious relationship with students, students tend to depend on their teachers, thinking that teachers will recognize the learning difficulties and tolerate their inertia, which ultimately negatively affects their self-efficacy.

### Practical Implications

According to our findings, this study has important practical significance for learning engagement among college students in the e-learning context. A close relationship between teachers and students, students’ autonomous control over class, and mutual support and referents among peers are considered predictive factors for self-efficacy, subjective well-being, and learning engagement. External environmental stimuli have an effect on psychological status and help students gain more positive inner feelings, so they can be regarded as an essential condition for improving student learning engagement. Teachers should focus on motivating students to engage in learning on their own initiative while asking them to achieve goals. We therefore make the following suggestions for long-term study at home during the global COVID-19 pandemic. First, teachers should be encouraged to grant students more control over their learning, provide a more active online teaching atmosphere, add relevant applied technologies, and enhance students’ sense of participation and control in class. Some studies have indicated that teachers play an important role in guiding students to control their learning (Zimmerman et al., 1996). Thus, schools should provide teachers with brief training courses on how to promote these techniques in e-learning.

Second, teachers should support communication among students in the e-learning environment. Students are prone to be affected by the ideas of their peers, so teachers should increase opportunities for communication among students, creating a learning and social environment that is conducive to relationship maintenance and strengthens interaction. Moreover, providing more interfaces and functions for interaction and communication among students can also be introduced to the online teaching platform. Our findings not only can enrich the research on student interaction in e-learning but also can help teachers and platforms that provide online courses in the future.

Third, teachers should develop good teacher–student relationships. This study suggests that teachers also need to state course requirements and objectives clearly to students while creating a close relationship to reduce environmental barriers. Teachers should pay close attention to the subtle changes in the teacher–student relationship in the network environment and observe the mental and learning states of students while teaching. Teachers should have a definite attitude toward student inertia and contain the development of adverse mental states in a timely manner.

### Research Limitations

Our research findings will enrich the literature on learning engagement, the S-O-R model, and e-learning environments. Nevertheless, some limitations exist and represent further research directions. First, although the S-O-R model has achieved a remarkable position in the field of psychology, only a few studies have focused on the relationship between stimulating factors in the e-learning environment and learning engagement among college students. This study builds the constructive mechanism for learning engagement in the e-learning environment based on the S-O-R model (perceived closeness, perceived control, and peer referents) and extracts important learning theories, but considering the unique, long-term, and large-scale e-learning environment generated by the COVID-19 pandemic, future research should test the model under different scenarios, such as e-learning as a supplement to face-to-face instruction.

Second, the data used in this study came from courses for a medium number of students (40–70 students), and no typical large-scale courses for audiences in the hundreds were involved. Thus, it remains to be seen whether student control over courses, harmonious teacher–student relationships, and extensive peer referents in e-learning for a larger number of students can bring similar benefits.

Third, the sample in this study may not accurately represent all student groups due to the restrictions of time and space. Thus, future research should include and compare different ethnic and cultural groups to provide additional opinions on e-learning, in addition to expanding the sample size and improving the research representativeness.

## Data Availability Statement

The raw data supporting the conclusions of this article will be made available by the authors, without undue reservation.

## Ethics Statement

The studies involving human participants were reviewed and approved by Institutional Review Board, Yango University. The patients/participants provided their written informed consent to participate in this study.

## Author Contributions

This study is a joint work of the four authors. JY and MP contributed to the ideas of educational research, collection of data, and empirical analysis. MP, SW, and WC contributed to the data analysis, design of research methods, and tables. MP, SW, WC, and JY participated in developing a research design, writing, and interpreting the analysis. All authors contributed to the literature review and conclusions.

## Conflict of Interest

The authors declare that the research was conducted in the absence of any commercial or financial relationships that could be construed as a potential conflict of interest.
